# In vitro pre-vascularized model of the human skin based on collagen hydrogel reinforced by fibrin-coated nanofibrous membrane

**DOI:** 10.1177/20417314261478553

**Published:** 2026-08-31

**Authors:** Julia Tomsu, Martina Doubkova, Antonin Broz, Andreu Blanquer, Daniel Hadraba, Martina Travnickova, Hubert Suca, Monika Supova, Vera Jencova, Lucie Bacakova

**Affiliations:** 1 Laboratory of Biomaterials and Tissue Engineering, 86869 Institute of Physiology, Czech Academy of Sciences, Prague, Czech Republic; 2 Departament de Biologia Cel·lular, Fisiologia i Immunologia, 16719 Universitat Autònoma de Barcelona, Bellaterra, Spain; 3 Laboratory of Advanced Microscopy and Data Analyses, 86869 Institute of Physiology, Czech Academy of Sciences, Prague, Czech Republic; 4 Prague Burn Center, Third Faculty of Medicine, Charles University and Královské Vinohrady University Hospital, Prague, Czech Republic; 5 Institute of Rock Structure and Mechanics, 86869 Czech Academy of Sciences, Prague, Czech Republic; 6 Department of Chemistry, Faculty of Science, Humanities and Education, 496042 Technical University of Liberec, Liberec, Czech Republic

**Keywords:** skin model, 3D vascular network, collagen, hydrogel, co-culture, fibrin, nanofibrous membrane, biodegradable polyesters, air-liquid interface, tissue engineering

## Abstract

Introducing pre-vascularization in functional full-thickness skin equivalents is critical to improve inosculation upon implantation. This study introduces a mechanically reinforced, bi-layered human skin model comprised of epidermis and pre-vascularized dermis. By combining a collagen type I hydrogel with an electrospun PCL/PLCL nanofibrous membrane coated with fibrin provisional matrix, we developed a composite scaffold that prevents the cell-induced hydrogel contraction and replicates the mechanical heterogeneity of native skin. The model utilizes a simultaneous tri-culture of human endothelial cells, adipose tissue-derived stromal cells, and keratinocytes. A custom 3D-printed, adjustable insert for cultivation at an air-liquid interface facilitates stratification of the dermal and epidermal layers. Our results demonstrate that adipose tissue-derived stromal cells effectively function as pericyte-like stabilizers, driving the formation of dermal capillary-like networks in 3D. This reinforced, pre-vascularized model provides a tool for improving tissue substitutes *in vitro* and *in vivo*, and a biomimetic platform for studying dermal-epidermal crosstalk and microvascular formation.

## Introduction

Human skin is a multifunctional organ that forms the largest and most exposed interface between the body and the environment. It serves as a protective barrier against mechanical damage, microbial invasion, and water loss, while also participating in thermoregulation, sensory perception, and immune response.^1^ Structurally, the skin consists of three primary layers: the epidermis, composed mainly of stratified keratinocytes undergoing continuous renewal and terminal differentiation; the dermis, a vascularized connective tissue matrix populated by fibroblasts, endothelial cells, and immune cells, which is enriched with extracellular matrix (ECM) components such as collagen types I and III; and the hypodermis, a highly vascularized layer constituted mainly of subcutaneous fat.^2^ The presence of a functional dermal vascular network is essential for nutrient and oxygen supply, especially for maintaining a healthy, proliferative basal layer of the epidermis. In the context of tissue engineering and regenerative medicine, skin substitutes have attracted considerable attention for their potential to restore skin function after injury, particularly in cases of full-thickness burns, chronic wounds, or surgical defects.^3^ Moreover, *in vitro* skin models are increasingly used as alternatives to animal testing for evaluating drug delivery, cosmetic safety, and disease modeling, including conditions such as psoriasis, atopic dermatitis, or melanoma,^4,5^ or pathological wound healing, e.g., hypertrophic and keloid scars.^6^ Various types of engineered skin models exist, ranging from simple epidermal equivalents, consisting only of keratinocytes cultured on a planar surface, to full-thickness skin equivalents that incorporate both dermal and epidermal components,^7^ and even a hypodermal layer.^8^ However, a major limitation of many existing constructs is the absence of functional vasculature, which restricts the size and longevity of the graft, hinders nutrient diffusion, and limits the physiological relevance of the model.^9^ Strategies to overcome this limitation include pre-vascularization – the *in vitro* formation of microvascular networks before grafting – introducing endothelial cells, angiogenic factors, and biomimetic scaffolds like hydrogels in co-culture with fibroblasts and keratinocytes,^9^ or building a construct by compressing together separately pre-cultured layers composed of different cell types.^8^

In this study, we report the development of a pre-vascularized, bi-layered skin model that combines biomimetic architecture with a simultaneous tri-culture of skin-relevant cells. The model was based on a collagen type I hydrogel mechanically reinforced by an electrospun nanofibrous membrane composed of a blend of poly(ε-caprolactone) (PCL) and poly(L-lactide-co-ε-caprolactone) (PLCL). To enhance cell adhesion and mimic the provisional matrix of wound healing, the membrane was coated with fibrin, a natural polymer derived from fibrinogen polymerization, known for its angiogenic and cell-supportive properties.^10^ Adipose tissue-derived stem cells (ADSCs) were seeded onto the surface of the fibrin-coated nanofibrous membrane to support dermal regeneration through paracrine signaling and potential transdifferentiation.^11^ A collagen hydrogel cast on top of the membrane served as a soft, ECM-like material for encapsulating human umbilical vein endothelial cells (HUVECs), thereby promoting the formation of early capillary-like networks. Primary human keratinocytes (hKs) were seeded on top of the collagen-HUVEC layer to form the epidermal compartment. To promote proper stratification and terminal differentiation of keratinocytes, the model was cultured at an air-liquid interface (ALI), a critical step in generating functional epidermal equivalents.^12^ To facilitate controlled layering and ALI culture, we developed a custom-made 3D-printed insert system for a 6-well plate with adjustable height, enabling fine control over the epidermal layer’s exposure to air.^13^ The pre-vascularized skin model was evaluated using confocal microscopy, image analysis, and mechanical testing.

## Materials and methods

### 3D-printed air-liquid interface system

**Figure 1.**
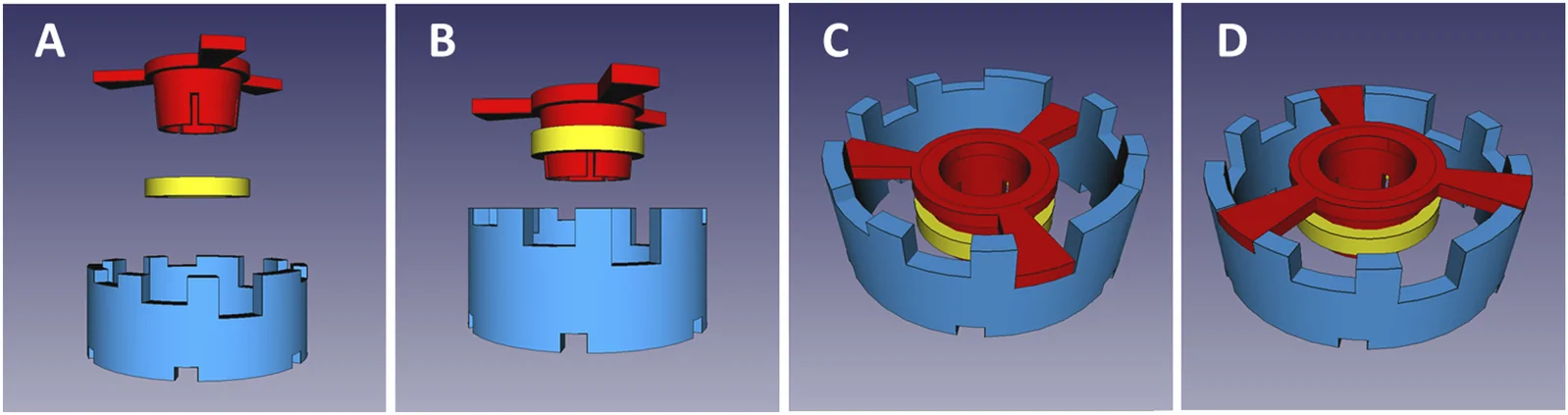
3D-printed model of the air-liquid insert designed to hold the membranes that support the skin construct: (a) Separated insert (red), fixing ring (yellow) and holder (blue). (b) Fixation of the membrane on the insert with the fixing ring. (c) Position for submerged cultivation. (d) Position for cultivation at the air-liquid interface (ALI).

A customized 3D-printed crown holder insert was developed as support for skin construct preparation and keratinocyte differentiation. The inserts fit into the 6-well plate (TPP, Switzerland). The position of the inserts inside the well plate, i.e. the distance from the bottom of the 6-well plate, was made adjustable by changing the position of the insert with affixed membrane in the outer ring of the holder. Moving the insert up in the holder to the uppermost position exposes the cultured hydrogel model to the air-liquid interface (ALI) (Figure 1). The ALI aims to mimic *in vivo* conditions in which the keratinocytes grow, enabling their stratification. The volume of medium used for skin construct culture was 5 mL. This enabled the cells to grow for a longer period without changing the medium and exposing the model to the ALI.

### Nanofibrous membranes

A nanofibrous membrane was prepared from a blend of poly(ε-caprolactone) (PCL) and poly(L-lactide-co-ε-caprolactone) (PLCL). Nanofibres were prepared using DC needleless electrospinning technique (Nanospider 1WS500U, Elmarco, Liberec, Czech Republic). The electrospinning solution contained a mixture of 5% (w/w) PCL (PCL; Mn 80,000 g/mol; Merck, Darmstadt, Germany) and 5% (w/w) PLCL (Purasorb PLC 7015, Corbion, Amsterdam, Netherlands) in chloroform/ethanol/acetic acid solution (8:1:1). The electrospinning itself was performed the same way and at the same conditions as described in Blanquer et al.^14^ and led to the fabrication of a membrane with the same parameters (i.e. diameter of most fibres was in the range of 400 to 800 nm). Membrane structure was visualized by Scanning Electron Microscopy (Vega 3SB Easy Probe; TESCAN, Czech Republic). Before the experiments, the membranes were cut into squares (approx. 1.7 x 1.7 cm), fixed in the customized 3D-printed crown holders, and inserted into 24-well plates (Techno Plastic Products AG, Switzerland). Subsequently, they were disinfected with 70% ethanol (1 hour), rinsed with deionized water (15 min), and sterilized with UV light (30 min).

### Fibrin mesh nanocoating

The fibrin nanocoating on the nanofibrous membranes was formed by the activation of water-soluble human fibrinogen (341576; EMD Millipore, USA) with human thrombin (T6884, Sigma-Aldrich, USA). The fibrin nanocoating procedure was described in detail in our earlier study by Pajorova et al.^15^ Briefly, in the first step, fibrinogen at a concentration of 10 µg/mL in trisaminomethane (Tris) buffer (50 mM Tris-HCl, 100 nM NaCl, and 2.5 mM CaCl_2_) was applied on the membrane for 1 hour. The unreacted fibrinogen was washed away with pure Tris buffer. In the second step, the adsorbed fibrinogen was activated with thrombin (2.5 U/mL in Tris buffer) for 15 minutes, and the unreacted thrombin was aspirated. Then, a solution of 200 µg/mL of fibrinogen in Tris buffer and 0.5 U/mL of antithrombin III (820720, Chromogenix, Italy) in deionized water was added to the membranes for 1 hour to form a stable fibrin mesh.

The morphology of the fibrin nanocoating was visualized by immunofluorescence staining. The nanocoated nanofibrous membranes were blocked with 1% bovine serum albumin (BSA; Sigma-Aldrich Co., USA) in phosphate-buffered saline (PBS), followed by treatment with 1% Tween (Sigma-Aldrich Co., USA) in PBS. The samples were incubated with rabbit polyclonal anti-human fibrinogen antibody (A0080, Dako Denmark A/S, Denmark) diluted 1:200 in PBS overnight at 4°C. The samples were then incubated with goat anti-rabbit antibody conjugated with Alexa Fluor 488 (A11070, Thermo Fisher Scientific, USA) diluted 1:400 in PBS for 1 hour in the dark at room temperature.

### Collagen hydrogel preparation

Hydrogels were prepared using type I collagen (COL) isolated from porcine skin according to the protocol described by Filova et al.^16^ Briefly, the first step involved incubation of cut skin in 70% (v/v) ethanol solution (1 g of skin per 10 mL) for 30 min. The skin was then washed with water and subsequently exposed to an acetic acid solution (1:1000, v/v) at a ratio of 1 g of skin per 20 mL for 48 hours at 4°C. Collagen in supernatant was precipitated using 0.1 M NaOH at a ration of 6:1 (v/v) until pH 7 was reached. The obtained pellets were then dissolved in acetic acid solution (1:1000, v/v), frozen at -30°C and lyophilized. The lyophilized COL was dissolved in 0.02 M acetic acid to a concentration of 5 mg/mL and stored at 4°C for 5 days. The COL suspension was then homogenized using a disintegrator (10,000 rpm, 10 min). The hydrogel was used for the construction of the bi-layered pre-vascularized skin model (see below).

### Cell isolation, characterization, and culture conditions

*Human adipose tissue-derived stem cells* (ADSCs) were isolated from subcutaneous abdominal adipose tissue obtained by liposuction from a healthy female donors (age 38.3 ± 8.6 years) at the Department of Plastic Surgery, Na Bulovce Hospital and First Faculty of Medicine, Charles University, Prague, after approval of the Ethics Committee (No. 16.3.2023/107 95/EK-Z) and informed consent of the patient, in compliance with the Declaration of Helsinki. The isolation procedure was performed as described in our previous article.^17^ In brief, the fresh lipoaspirate was washed several times with PBS, and then it was digested using type I collagenase 0.1% (w/v) (LS004214, Worthington Biochemical Corp., USA) diluted in PBS with 1% (w/v) BSA. After the one-hour enzymatic digestion, the lipoaspirate was centrifuged, and the stromal vascular fraction (SVF) was separated from the rest of the digested tissue. SVF was washed three times, filtered using a cell strainer (pore size 100 µm), and seeded into culture flasks (75 cm^2^, TPP, Switzerland) at a density of 12 mL of original lipoaspirate/flask. The isolated cells were cultured in DMEM (Gibco, USA), supplemented with 10% (v/v) FBS (Gibco, USA), 40 µg/mL of gentamicin (LEK, Slovenia), and 10 ng/mL of recombinant human basic fibroblasts growth factor (FGF2; GenScript, USA). The primary cells in passage 0 were frozen after reaching 80% confluence, and the remaining cells were subcultured. Subsequently, the cells in passage 2 were characterized by flow cytometry (Accuri C6 Flow Cytometer, BD Biosciences, USA) using FITC-, Alexa488-, Alexa647-, or PE-conjugated monoclonal antibodies against specific CD markers. For our experiments, we selected a single donor’s ADSC primary cultures with the highest CD146 positivity (76.1%; laminin alpha 4 receptor), which is also considered a marker of high angiogenic potential and pericyte-like activity.^18–20^ These cells expressed standard ADSC surface markers, namely CD105 (99.8%; endoglin), CD90 (99.7%; immunoglobulin Thy-1), CD73 (99.8%; ecto-5′-nucleotidase), CD29 (99.8%; fibronectin receptor), and CD146 (76.1%). At the same time, the isolated ADSCs were negative or nearly negative for hematopoietic and endothelial markers, such as CD45 (7.1%; protein tyrosine phosphatase receptor type C), CD34 (0.3%; antigen of hematopoietic progenitor cells), and CD31 (0.2%; platelet-endothelial cell adhesion molecule-1). The cells were used in passages 1–3 and cultivated at 37°C in a humidified atmosphere with 5% CO_2_.

*Human umbilical vein endothelial cells* (HUVECs) were purchased from Lonza (Switzerland). The cells were cultured in the endothelial cell growth medium 2 (EGM-2), which was prepared from the endothelial cell basal medium 2 (PromoCell, Germany) supplemented with 0.2 µg/mL of hydrocortisone, 22.5 µg/mL of heparin, 1 µg/mL of ascorbic acid, 5 ng/mL of EGF, 0.5 ng/mL of vascular endothelial growth factor (VEGF) 165, in an isoform of (VEGF-A), 20 ng/mL of insulin-like growth factor (IGF-1), 10 ng/mL of FGF2 and 2% of FBS (PromoCell, Germany), and 1% of antibiotic-antimycotic solution (Penicillin-Streptomycin-Amphotericin B Suspension; A5955, Sigma-Aldrich, USA; 100x concentrated).

*Primary human keratinocytes* (hKs) were isolated from the skin of a young adult donor at the University Hospital Kralovské Vinohrady, after obtaining approval of the Ethics Committee (Third Faculty of Medicine, Charles University, approval number EK-VP/15/0/2017) and informed consent of the patient, in compliance with the Declaration of Helsinki. The epidermis and dermis were separated by enzymatic digestion using neutral protease (LS02104, Dispase, Worthington Biochemical Corp., USA). Single keratinocytes were isolated from the epidermis using dynamic trypsinization as described by Wang et al.^21^ Keratinocytes were seeded onto a feeder layer of 3T3 mouse embryonic fibroblasts previously inactivated using mitomycin C (M0503, Sigma-Aldrich, USA) and cultivated in a mixture of the DMEM medium and Ham’s Nutrient Mixture F12 medium (DMEM/F12) in a ratio of 3:1. The mixture of the media was supplemented with 10% of FBS, 10 ng/mL of EGF, 10 µg/mL of insulin, 0.4 µg/mL of hydrocortisone, 10^−10^ M of cholera toxin and 1% of antibiotic-antimycotic solution (all from Sigma-Aldrich, USA). Primary keratinocytes were used in passages 1–3 and cultivated at 37°C in a humidified atmosphere of 5% CO_2_.

### The construction of a bi-layered pre-vascularized skin model

The PCL/PLCL nanofibrous membranes fixed in the ALI system and coated with fibrin were seeded with ADSCs at a density of 30,000 cells per sample in DMEM supplemented with 10% FBS, 10 ng/mL of FGF2 and antibiotics. After 3 days, the cultivation media were aspirated and the collagen hydrogels were cast onto ADSC-preseeded membranes, as described in our earlier study.^22^ However, the type I collagen used in the present study was isolated from porcine skin and polymerized either with or without HUVECs homogeneously dispersed within the hydrogel. Briefly, the 390 µL of EGM-2 medium with all supplements described above was prepared with or without HUVECs and mixed with 600 µL of COL suspension (5 mg/mL), and 10 µL of 7.5% sodium bicarbonate. The final concentration of COL in hydrogels was 3 mg/mL and the cell density was 1,600,000 cells/mL of the hydrogel. A total volume of 500 µL of the hydrogel mixture was applied by micropipette onto each ADSC-preseeded membrane and was allowed to polymerize for 30 minutes at 37°C, in a humidified atmosphere with 5% CO_2_. Then, 5 mL of EGM-2 medium with all supplements was added to each sample.

**Figure 2.**
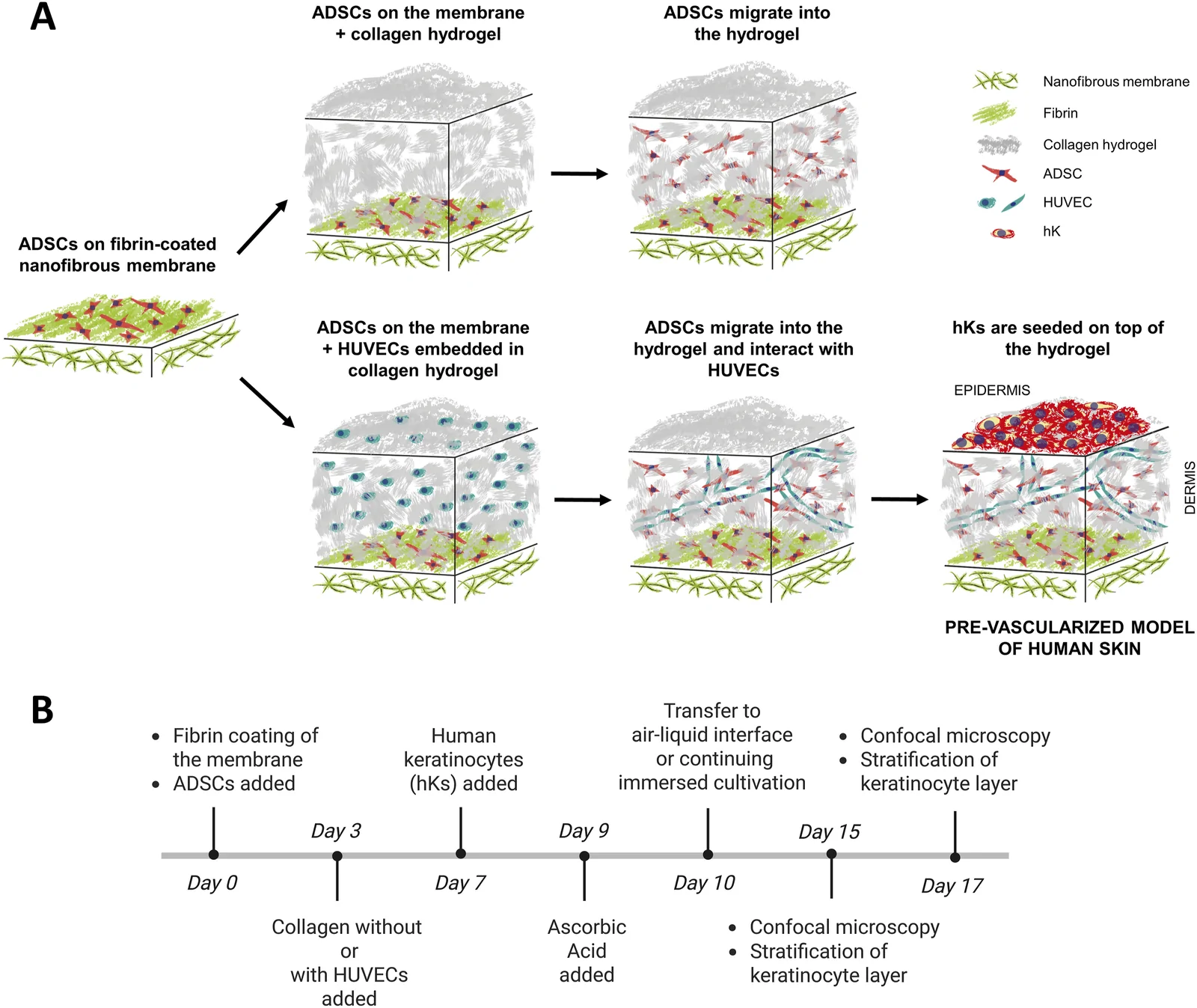
Preparation of the pre-vascularized bi-layered skin model. (a) The model consists of a fibrin-coated PCL/PLCL membrane seeded with ADSCs, collagen hydrogel with or without embedded HUVECs, and hKs seeded on the surface of the hydrogel. The keratinized layer of hKs on top serves as the epidermis. The collagen hydrogel, populated with a co-culture of ADSCs and HUVECs that form capillary-like structures through mutual interactions, serves as the dermis. (b) Scheme of the experimental workflow.

At day 7 of the experiment, the primary hKs were seeded onto the hydrogel group containing HUVECs at a density of 100,000 cells/sample in EGM-2/KGM (1:1; PromoCell, Germany; Lonza, Switzerland). The medium was replaced after 2 days with fresh EGM-2/KGM (1:1) media mixture and 50 μg/mL of ascorbic acid (2-phospho-L-ascorbic acid trisodium salt, 49752, Sigma-Aldrich, USA). The medium with ascorbic acid was changed after 4 days. On day 10 of the experiment, half of the samples were positioned above the surface of cultivation medium to stimulate stratification of keratinocytes growing at the ALI (i.e., immersed or ALI group). The final model was approximately 0.5 mm thick. The skin model preparation process and the experimental workflow are described in Figure 2.

In the models containing only ADSCs or only ADSCs and HUVECs (showed to provide comparison with the completed tri-culture model), the workflow was the same as described above, with minor changes in respect to the cell types: In ADSC-only model, the hydrogel without cells was cast on top of the ADSC-preseeded membrane and cultivated up to day 17 in DMEM supplemented with 10% FBS, 10 ng/mL of FGF2 and antibiotics, changed every 4 days. In the ADSC+HUVEC-only model, the hydrogel containing HUVECs was cast on top of the ADSC-preseeded membrane, the medium was replaced with EGM-2 with all supplements, and the cells were cultivated up to day 17, with medium changes every 4 days.

### Characterization of the skin model

The migration of the ADSCs into the collagen hydrogels and their interactions with HUVECs were evaluated using fluorescent staining after 17 days of cultivation without keratinocytes. The interactions among all three cell types in the collagen construct were also evaluated after 17 days.

#### Cell migration into collagen hydrogel and cell morphology

The adhesion, spreading, and morphology of the cells were visualized by staining with a combination of fluorescent dyes. Before staining, the cells were fixed with 4% paraformaldehyde for 30 min. The cells were permeabilized with 1% Tween 20 diluted in PBS for 1 hour at room temperature. The non-specific binding sites were blocked using a solution of 1% BSA diluted in 0.1% Triton X-100 in PBS (all from Sigma-Aldrich Co., USA), also for 1 hour at room temperature. Phalloidin conjugated with fluorescent dyes, i.e. with tetramethylrhodamine B isothiocyanate (TRITC) (5 µg/mL; P1951; Sigma-Aldrich, USA) or Atto 488 (0.04 nmol/mL; 49409; Sigma-Aldrich Co., USA), was used for actin filaments staining. Staining with 4′,6-diamidino-2-phenylindole (DAPI; 1 µg/mL; Sigma-Aldrich Co., USA) was used for cell nuclei. All antibodies were diluted in the same blocking solution as described above at 1:400 and incubated with samples for 1 hour in the dark at room temperature.

#### Capillary-like network formation

The ADSCs and HUVECs were co-cultured in 3D collagen hydrogels for 17 days to initiate capillary-like network formation. Before the fluorescent staining, the hydrogels were separated from the underlying membranes. CD31, a specific marker of endothelial cells, was stained with primary and secondary antibodies after fixation with 4% paraformaldehyde, permeabilization, and blocking as described above. The primary antibody to human CD31 (11-273-C100, mouse monoclonal, clone MEM-05; EXBIO antibodies, Czech Republic) was diluted in blocking solution (1% BSA and 0.1% Tween 20 in PBS) at a 1:200 ratio and incubated with the samples for 1 hour at 37°C. The secondary anti-mouse antibody conjugated to Alexa Fluor 633 (A-21053; Thermo Fisher Scientific, USA) was diluted 1:400 in blocking solution and incubated with the samples for 1 hour in the dark at room temperature. The actin filaments in the cells were stained with phalloidin conjugated with Atto 488 fluorescent dye (0.04 nmol/mL; 49409; Sigma-Aldrich Co., USA), and the cell nuclei with DAPI (1 µg/mL; Sigma-Aldrich Co., USA).

#### Keratinocyte layer stratification

In order to visualize the stratification of keratinocytes, the immunofluorescence staining of cytokeratin 14 (CK14) and cytokeratin 10 (CK10) was performed. The primary antibodies against human CK14 (ab7800, mouse monoclonal, clone LL002; Abcam, UK) were used for the staining of the basal keratinocytes in the lower layers of the forming epidermis, while the primary antibody against human CK10 (ab76318, rabbit monoclonal, clone EP1607IHCY; Abcam, UK) was used for the staining of the differentiated keratinocytes in the upper layers of stratified epidermis. All primary antibodies were diluted in blocking solution (1% BSA and 0.1% Tween 20 in PBS) at a 1:200 ratio and incubated with samples for 1 hour at 37°C. The secondary antibodies, namely anti-rabbit or anti-mouse antibodies conjugated to Alexa Fluor 488 (A-11070; Invitrogen, Thermo Fisher Scientific, USA) or Alexa Fluor 546 (A-110003; Invitrogen, Thermo Fisher Scientific, USA), were used according to the primary antibody combination. All secondary antibodies were diluted in the same blocking solution as described above at 1:400 and incubated with samples for 1 hour in the dark at room temperature.

### Confocal microscopy

All images visualizing immunofluorescence staining of the cells were acquired using the Andor Dragonfly 503 (Andor, UK) spinning disk confocal system mounted on an inverted microscope Leica DMi8 (Leica, Germany), controlled by Fusion software (Andor Technology Ltd, Belfast, UK), using following band-pass filters: DAPI 450/50, A488/FITC 525/50, TRITC 600/50, Cy5 700/75. The images were used to assess cell interactions and their ability to migrate through the hydrogel, to measure the thickness of the keratinized layer on top of the model, and to measure the percentage area of the top layer covered by differentiated keratinocytes. Images for presentation in this article were generated from confocal 3D stacks using Imaris software (Bitplane, Belfast, UK) in 3D View and Section View. To enhance image clarity, all images that were used for the figures were deconvolved using the Huygens Essential 21.04 (Scientific Volume Imaging) built-in standard deconvolution. The color coding used in the presented images was selected to provide clear visual representation and does not necessarily correspond to the florophore used.

### Analysis of fluorescence images

Microphotographs obtained from the confocal microscope were analysed to describe the keratinocyte layers formed on top of the 3D collagen hydrogel construct after 5 and 7 days of either additional cultivation in the immersed conditions or at the air-liquid interface (ALI); i.e. on day 15 and day 17 of the experiment.

The area covered by differentiated keratinocytes (i.e. positive for CK10 staining) was measured across at least three different regions of three parallel samples. The microphotographs were quantified using ImageJ FIJI software (v.1.54f), and the data is presented as a mean percentage of the total area of the top view image that was positive for CK10.

The thickness of the surface keratinocyte layer mimicking the epidermis was determined by measuring the multilayer of keratinocytes in 3D z-stack images from three regions and three parallel samples. Using the slice view option in Imaris software (Bitplane, Belfast, UK) the start and end point position of the keratinocyte layer was determined in the z-stack. To quantify the layer thickness in micrometers, the number of steps in the z-stack between the start and end positions was multiplied by the set value of the z-step size (i.e., the distance between acquired z-stack images). For this reason, in the images where the 3D volume data of the z-stack were visualized using Imaris Section View option to show orthogonal cross-sections, the displayed scale bar applies only to the XY plane (top view optical section), and not to the XY plane (front view) and YZ plane (side view) through the z-stack.

### Mechanical properties of the hydrogel construct

Hydrogel constructs without cells were measured to assess their mechanical properties as a scaffold using Atomic Force Microscopy (AFM) in Force Spectroscopy mode. Force spectroscopy measurements were taken after 17 days in cell culture media, including ALI cultivation. Each customized 3D-printed crown holder with the collagen construct was placed on the Petri dish, glued at the side of the crown, and watered for at least 30 minutes. Meanwhile, the JPK NanoWizard 3 AFM microscope (JPK, Berlin, Germany) was aligned for measurement in water, and after that, the CP-qp-CONT-SiO colloidal probe was calibrated (tip diameter 6.62 µm, cantilever spring constant 0.391 N/m, sensitivity 45.23 nm/V; NanoAndMore, Wetzlar, Germany). The total number of five samples was randomly probed at a minimum of eight locations per sample using force spectroscopy mapping. The single spectroscopy map size was 8 x 8 pixels at 25 µm^2^. This led to the acquisition of 64 curves for 8 randomly selected places for 5 samples, i.e. the sample size was 2560 independent force maps – Young’s moduli. The acquired curves were fitted using the Herz-Sneddon theory, and the Young’s modulus was established from the approaching part of the curve. The histogram of the acquired Young’s moduli showed distinct populations of values, as evidenced by the cluster analysis (k-means and elbow method). The data were clustered together using the aforementioned analysis and were plotted in a graph showing the Young’s moduli in an order of magnitude ranging from tens (Population 1) to hundreds (Population 2) to thousands (Population 3) of kPa.

### Statistical analysis

GraphPad Prism 10 (GraphPad Software, USA) was used to assess data normality and variance. Statistical analysis was performed using Two-Way ANOVA and Šidák’s multiple comparisons test, with p < 0.05 considered significant. Graphs were created in GraphPad Prism (GraphPad Software, Boston, MA, USA) and in MATLAB (Mathworks, Natick, MA, USA).

## Results

To investigate the potential of a bi-layered skin model combining stromal and endothelial components for pre-vascularization and epidermal stratification, we developed an *in vitro* model composed of adipose tissue-derived stromal cells (ADSCs), human umbilical vein endothelial cells (HUVECs), and human keratinocytes (hKs). ADSCs were first seeded on a fibrin-coated nanofibrous PCL/PLCL membrane and subsequently, after reaching confluence overlaid with a collagen hydrogel, either with or without embedded HUVECs. This setup enabled the formation of a pre-vascularized dermal-like layer. After initial co-culture, keratinocytes were seeded on top of the model and cultivated under either submerged or air-liquid interface (ALI) conditions to promote epidermal differentiation. Cellular behavior, including migration, morphology, and intercellular interactions, was evaluated using confocal microscopy and immunofluorescence staining over a 17-day cultivation period. The following sections describe the dynamics of ADSC migration, capillary-like structure formation, and keratinocyte stratification under the tested conditions.

**Figure 3.**
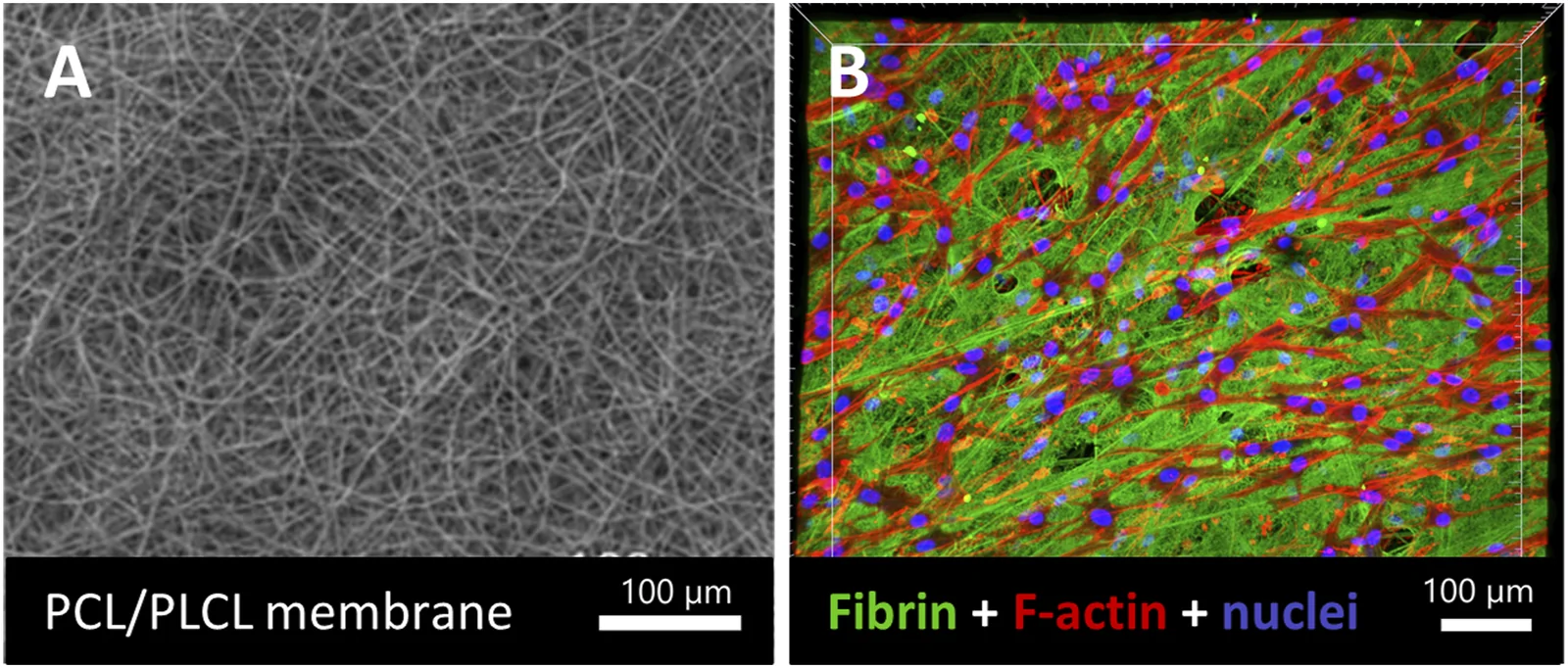
(a) The nanofibrous structure of the PCL/PLCL membrane visualized by Scanning Electron Microscopy (Vega 3SB Easy Probe (TESCAN, Czech Republic). Scale bar = 100 µm. The same membranes were also used in the study by Blanquer et al.^14^ (b) The morphology of fibrin nanocoating and ADSCs growing on the membrane visualized using fluorescence staining and confocal microscopy (fibrin – green; F-actin – red; nuclei – blue) on day 3 of the experiment, before the collagen hydrogel was cast on top. Andor Dragonfly 503 spinning disk confocal microscope. Scale bar = 100 µm.

**Figure 4.**
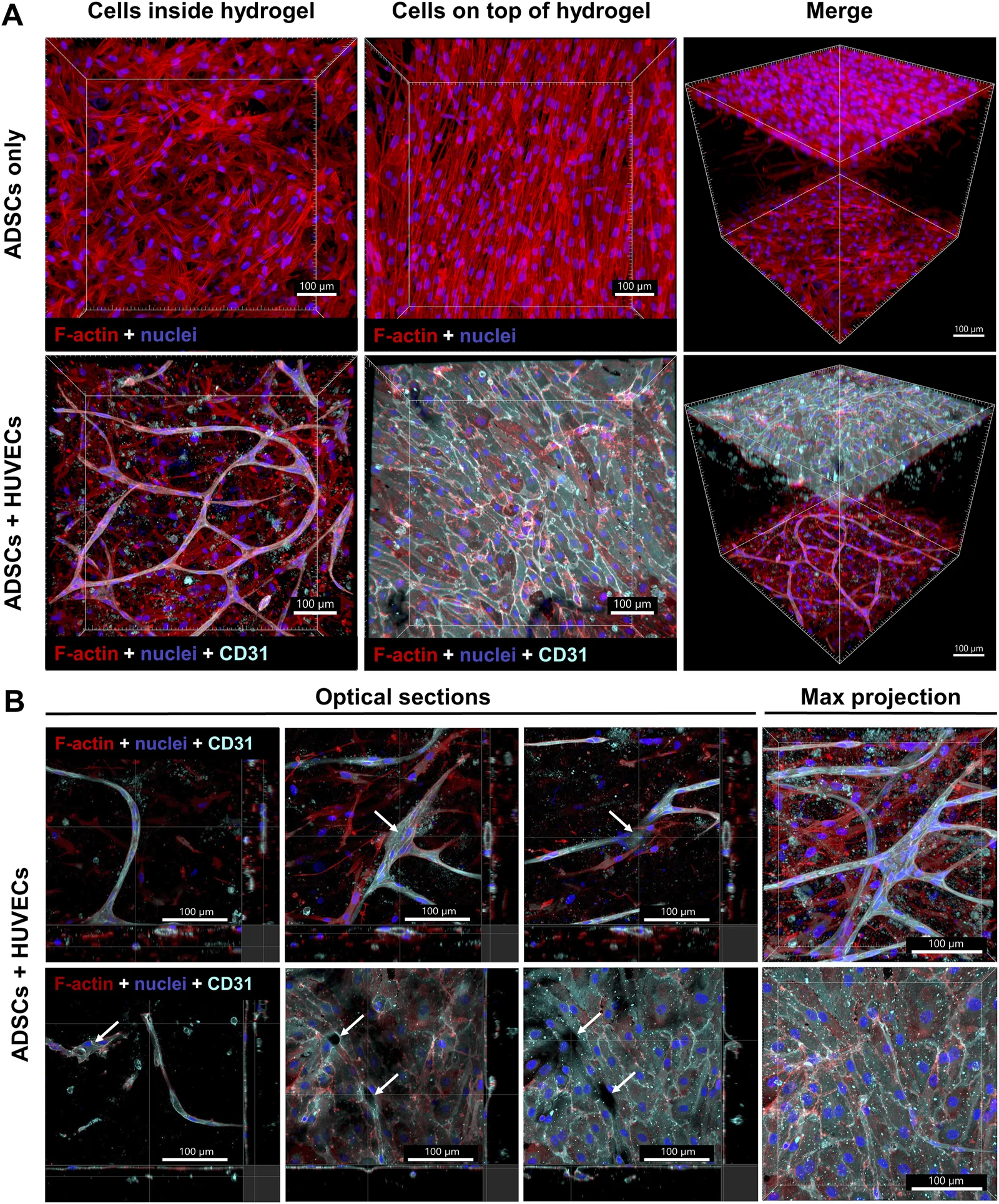
Preparation of the 3D pre-vascularized skin model – visualized at day 17 of culture: (a) Representative picture panel shows an inside view, a top view, and a merged 3D view of the hydrogels containing either ADSC-only culture or a co-culture of ADSCs and HUVECs. (b) Representative picture panel shows visualization of capillary-like structure network formation in the collagen hydrogel (first row), and the monolayer of HUVECs formed at the surface of the model without the addition of keratinocytes to the co-culture (second row). Both rows show a series of three optical sections along the XY plane (top view), and their corresponding orthogonal views in the XZ (front view), and YZ (side view) planes, together with a maximum projection of the z-stack. The images are arranged to display optical sections from the deepest layer to the top layer of the respective z-stack (left to right). White arrows indicate the void space inside the capillary-like structures (first row) and openings of the capillary-like structures on the hydrogel surface (second row). Color code matching the fluorescent staining is indicated in each image as follows: cytoskeletal F-actin (red), cell nuclei (purple-blue), CD31 membrane marker of HUVECs (light cyan). Andor Dragonfly 503 spinning disk confocal microscope, obj. 20× (a) or obj. 40× (b). Scale bar = 100 µm. The scale bar in the orthogonal views applies only to the top view (XY plane) optical section, not to the front and side views.

The ADSCs adhered and grew well on the nanofibrous membrane covered with fibrin (Figure 3). ADSC migration from the membrane into the hydrogel and throughout the hydrogel was observed after a 17-day cultivation period. When cultured without HUVECs or keratinocytes, ADSCs migrated extensively throughout the collagen hydrogel, even reaching its apical surface. The ADSCs displayed morphological changes that were influenced by their position in the hydrogel. During migration within the bulk collagen hydrogel, the ADSCs adopted a stellate morphology, with prolonged filopodia extending in multiple directions. Conversely, upon reaching the hydrogel surface and forming a confluent layer, their morphology changed to flattened, more elongated cells (Figure 4(a) – first row).

The introduction of HUVECs altered the migratory trajectory and positioning of the ADSCs, providing compelling evidence of their pericyte-like function. In the HUVEC co-culture, after 17 days of cultivation, the majority of ADSCs migrating from the fibrin-coated membrane remained within the hydrogel in close interaction with HUVECs, adopting a spindle-like morphology. This interaction resulted in the formation of capillary-like networks composed of both cell types, introducing pre-vascularization. Interestingly, the apical surface of the hydrogel was predominantly covered by HUVEC monolayer when the keratinocytes were absent (Figure 4(a) – second row).

Fluorescence microscopy also revealed that HUVECs tended to form a void space between them, especially inside the crossroads of capillary-like structures/tubes. These void spaces were observed continuing through several optical sections (Figure 4(b) – first row; see white arrows). In some areas, the monolayer of HUVECs on top of the hydrogel seemed to be interrupted by pits or openings with tubular character in the z-axis that were closely connected to the capillary branches located deeper in the collagen hydrogel (Figure 4(b) – second row; see white arrows). This image could be interpreted to mean that once the tubular pre-capillary structures reached the surface of the hydrogel, they opened onto it, and the endothelial cells subsequently spread into the squamous epithelium and formed a confluent layer.

**Figure 5.**
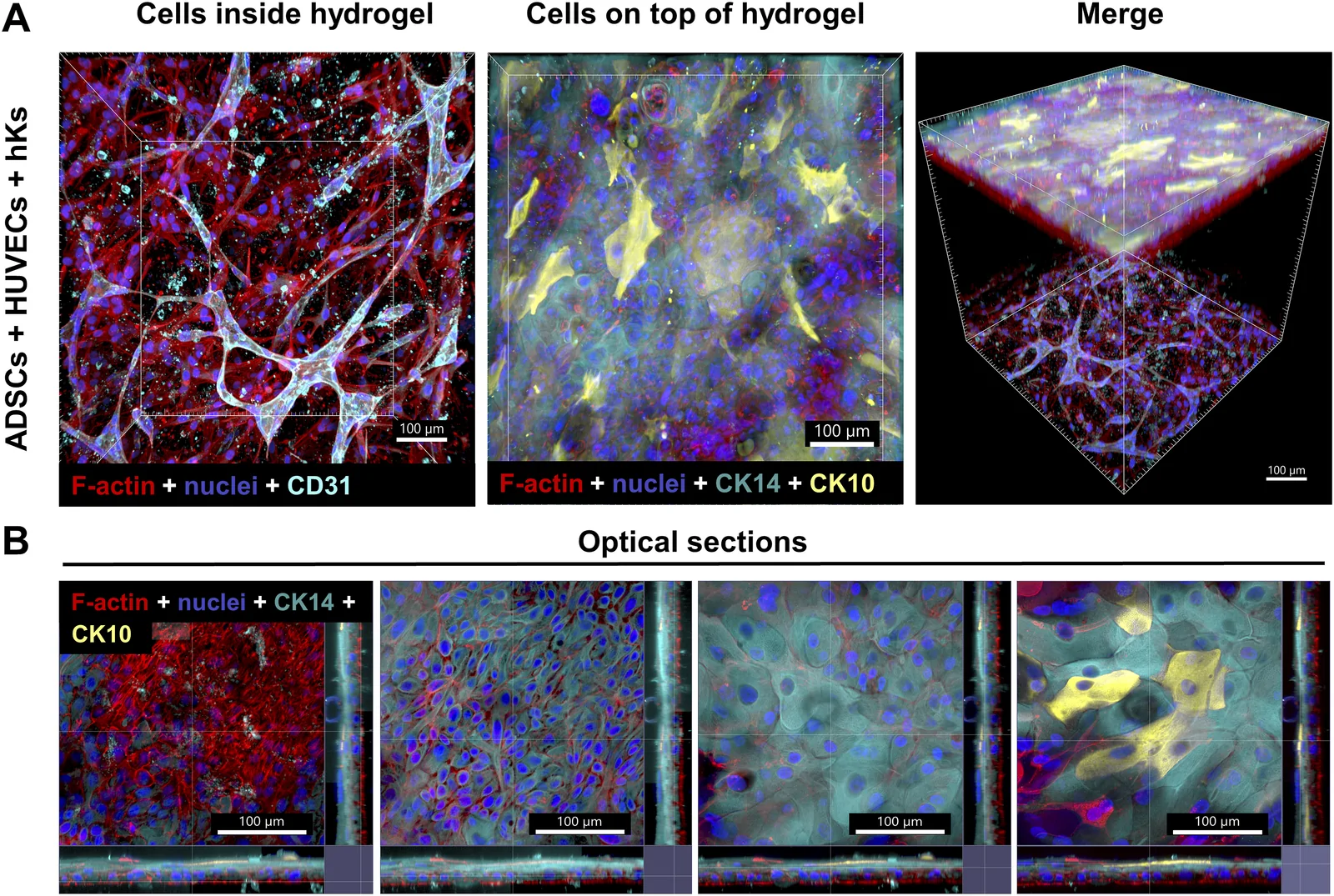
Final state of the 3D pre-vascularized skin model containing all three cell types – visualized at day 17 of culture: (a) Representative picture panel shows an inside view, a top view and a merged 3D view of the hydrogel containing a co-culture of ADSCs, HUVECs and keratinocytes (hKs). (b) Representative picture panel shows a visualization of the stratified layers of keratinocytes (hKs) on the hydrogel surface, presented as a series of four representative optical sections along the XY plane (top view), and their corresponding orthogonal views in the XZ (front view), and YZ (side view) planes. The images are arranged to display optical sections from the deepest layer to the top layer of the respective z-stack (left to right). Color code matching the fluorescent staining is indicated as follows: cytoskeletal F-actin (red), cell nuclei (purple-blue), CD31 membrane marker of HUVECs (light cyan), cytoskeletal CK14 in hKs (turquoise), and CK10, the late marker of differentiated hKs (light yellow). Andor Dragonfly 503 spinning disk confocal microscope, obj. 20× (a) or obj. 40× (b). Scale bar = 100 µm. The scale bar in the orthogonal views applies only to the top view (XY plane) optical section, not to the front and side views.

The final state of the model colonization was evaluated in a co-culture of all three cell types – i.e., the ADSCs were allowed to migrate from the nanofibrous membrane, the HUVECs were embedded in the collagen hydrogel, and hKs were added on top of the hydrogel on day 7 of the cultivation. Cell interactions were evaluated again on day 17 of the experiment, after 7 days of cultivation of the top layer of hKs at the ALI. Introduction of the third cell type, human keratinocytes (hKs), onto the surface of the hydrogel to develop the epidermal layer led to a subtle regression in the capillary-like structure formation, as compared to the initial ADSC/HUVEC co-culture; i.e., these structures did not reach the surface of the hydrogel and stopped growing beneath it. The interaction of HUVECs and ADSCs, however, still led to the formation of short branches and connections inside the hydrogel (Figure 5(a)). The hKs covered the entire surface of the hydrogel (Figure 5(a)), forming several stratified layers of cells, as shown by confocal microscopy optical sections that visualize cell morphology in various depths from the surface (Figure 5(b), left to right). A first confluent layer of small basal keratinocytes, identified by cytokeratin 14 staining, was located in direct contact with the surface of the collagen hydrogel, where strong actin cytoskeletal filaments were formed, as visualized by phalloidin staining. A second confluent layer, which consisted of larger keratinocytes, also expressing cytokeratin 14, was located directly above the layer of smaller keratinocytes and beneath a third, semi-confluent layer of differentiating suprabasal keratinocytes expressing cytokeratin 10. This observation demonstrates that after 7 days of cultivation at the air-liquid interface, the keratinocytes had already initiated differentiation, and the capillary-like structures remained stable.

The formation and stratification of the keratinocyte layer were evaluated by additional analyses on days 15 and 17 of the experiment, either after continuous cultivation in immersed conditions or after 5 and 7 days of cultivation at the air-liquid interface (ALI; starting on day 10), supporting the keratinization of the top layer. The cells in the collagen hydrogel were visualized by confocal microscopy to evaluate differences in the formation of pre-vascularization in the bottom layer and the state of the keratinocyte layer on top.

**Figure 6.**
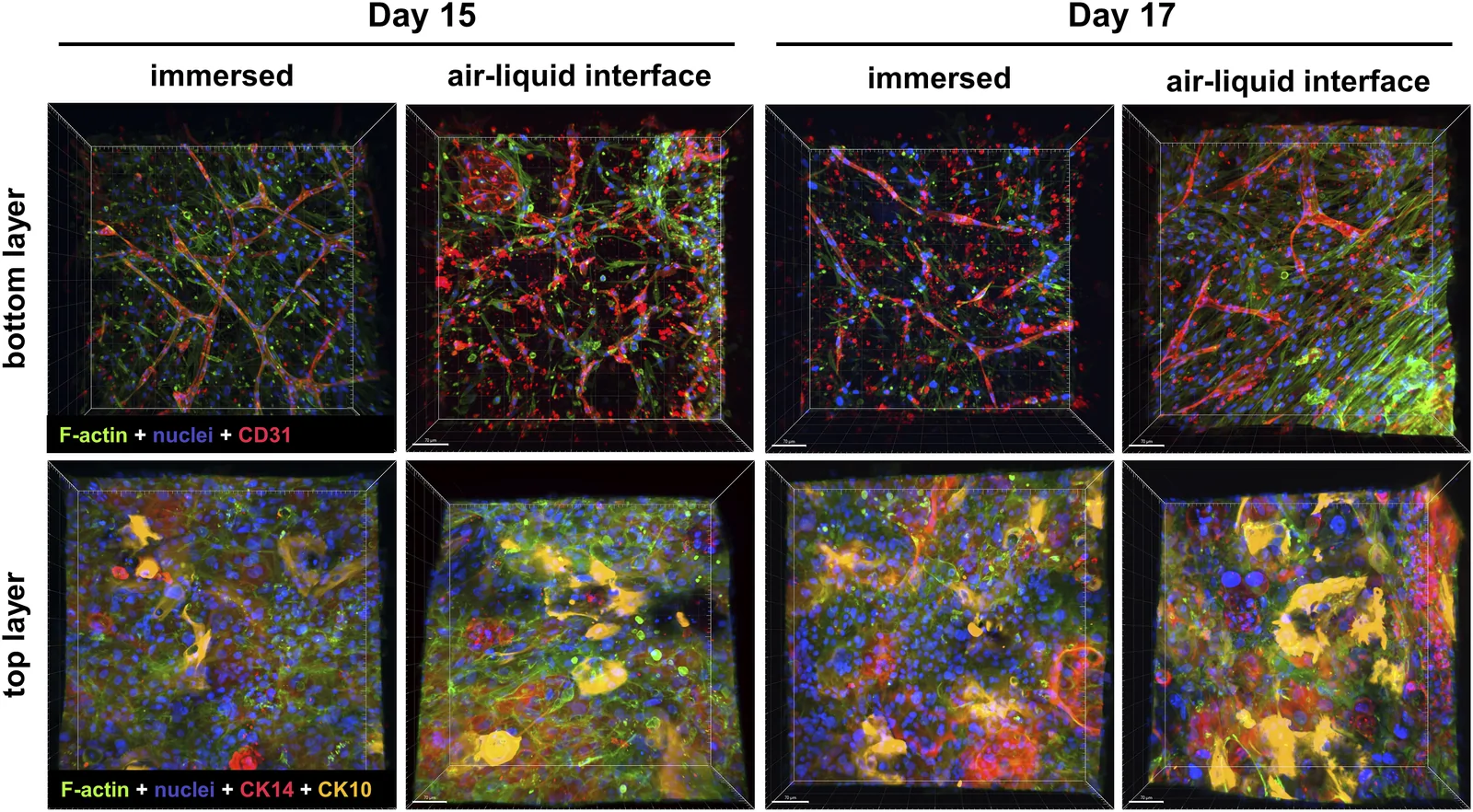
Adipose tissue-derived stromal cells (ADSCs), human umbilical vein endothelial cells (HUVECs) and human keratinocytes (hKs) co-culture. Cells co-cultured in collagen hydrogels after the seeding of keratinocytes on top of the model and additional culture under immersed and air-liquid interface conditions for 5 and 7 days (i.e. at day 15 and day 17 of the experiment). Images were captured from the bottom and top sides of the hydrogel. HUVECs were visualized using CD31 marker (red; bottom layer), basal keratinocytes using cytokeratin 14 (red; top layer) and differentiated keratinocytes using cytokeratin 10 (yellow; top layer) fluorescent staining. All cells were additionally stained for F-actin (green; bottom and top layer) and their nuclei (blue; bottom and top layer). Andor Dragonfly 503 spinning disk confocal microscope. Scale bar = 70 µm.

After 5 days (i.e., on day 15), results from the bottom layer showed that capillary-like structures were formed in both samples cultivated under immersed conditions and at the air-liquid interface (Figure 6, bottom layer). However, the structures appeared to be better developed in immersed samples. ADSCs were observed inside the collagen hydrogel and near the structured HUVEC cells. Fluorescence staining of cytokeratin 10 and cytokeratin 14 revealed an epidermal-like structure composed of multiple keratinocyte layers on top of the models. Several differentiated keratinocytes (cytokeratin 10-positive) were observed on both air-liquid interface and immersed samples (Figure 6, top layer), although a slightly higher number of differentiated cells was observed covering the top of the hydrogels cultivated at the air-liquid interface.

After 7 days (i.e., on day 17), capillary-like structures were formed in the bottom layer of both samples, regardless of cultivation conditions (immersed or in air-liquid interface; Figure 6, bottom layer). Again, the ADSCs were dispersed in collagen hydrogels near the structured HUVECs. Multiple layers of cytokeratin 14-positive keratinocytes were observed in most regions of the collagen surface. More differentiated cells (cytokeratin 10-positive) were growing on hydrogels cultivated in ALI conditions, covering a larger surface area (Figure 6).

**Figure 7.**
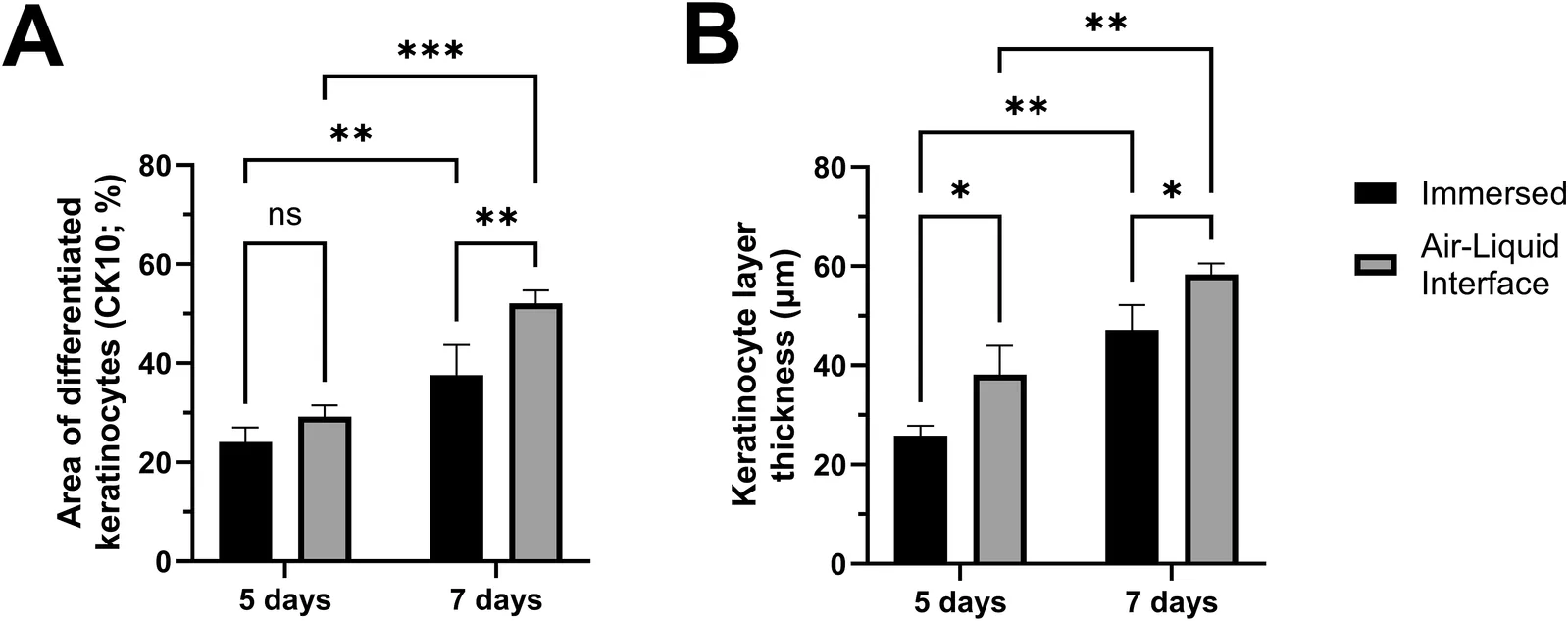
Analysis of the keratinocyte layer formed on top of the 3D collagen hydrogel construct after 5 and 7 days of additional cultivation in the immersed conditions or at the air-liquid interface (ALI); i.e. at day 15 and day 17 of the experiment: (a) Area covered by the differentiated keratinocytes positive for cytokeratin 10 (CK10) staining, and (b) thickness of the surface layer (mimicking epidermis) formed by the keratinocytes. The data are presented as mean with SD (n = 3). Two-Way ANOVA, Šidák’s multiple comparisons test, p < 0.05. Asterisks indicate the following statistical significances (respectively from left to right): (a) **p = 0.0032, **p = 0.0019, ***p = 0.0002; (b) *p = 0.0126, *p = 0.0206, **p = 0.0059, **p = 0.0073.

Subsequent image analysis showed that the percentage of area covered by differentiated keratinocytes in the immersed versus ALI samples was 23.13 ± 2.93 µm vs. 29.24 ± 2.27 µm on day 15, and 37.66 ± 6.05 µm vs. 52.08 ± 2.62 µm on day 17, respectively (Figure 7(a)). The overall thickness of the keratinocyte layer at the top of the hydrogels was generally higher in samples cultivated at the air-liquid interface and increased over time. Measurement showed that the full keratinocyte layer thickness on immersed versus ALI samples was 25.83 ± 2.02 µm vs. 38.17 ± 5.80 µm on day 15, and 47.17 ± 5.01 µm vs. 58.33 ± 2.26 µm on day 17, respectively (Figure 7(b)).

**Figure 8.**
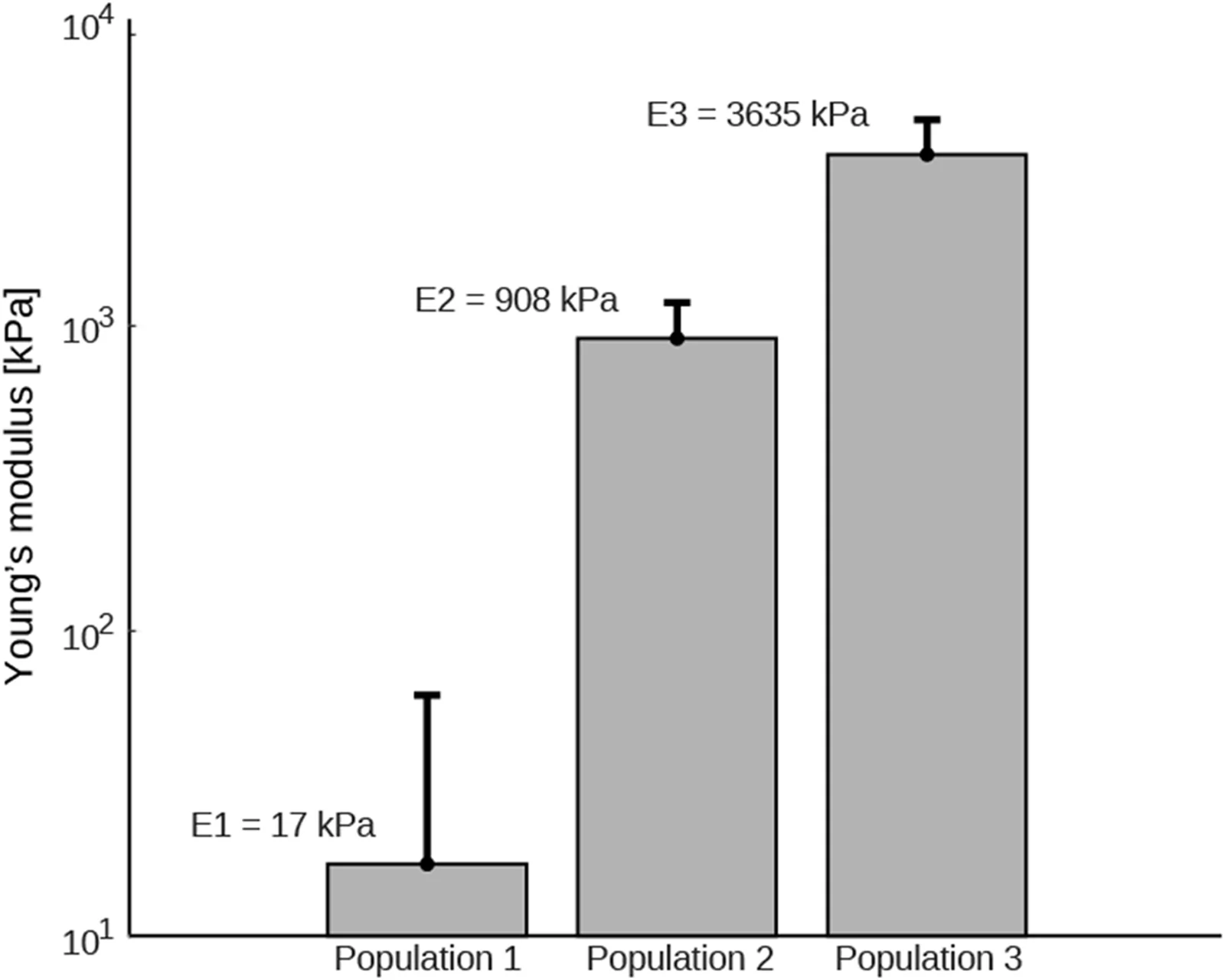
The Young’s moduli of the composite collagen construct. The Young’s modulus was obtained from the approached curves using the Herz-Sneddon model for a spherical probe. Three populations of Young’s modulus were clustered in the measured data.

Mechanical properties of the hydrogel constructs were measured after 17 days of cultivation including ALI, using samples without cells. Young’s moduli were obtained by AFM force probing, with the results demonstrating the composite nature of the scaffolds. Three distinct populations of Young’s moduli were identified in the samples (Figure 8) through K-means clustering and the elbow method. The mean ± SD values were as follows: Population 1 = 17 ± 44 kPa, Population 2 = 908 ± 274 kPa and Population 3 = 3607 ± 1102 kPa. The findings indicate the expected level of heterogeneity within the samples and reflect the composite structure of the scaffolds. The lowest modulus values are attributed to the collagen hydrogel, which typically exhibits mechanical properties in the kPa range. The intermediate Young’s modulus values likely correspond to regions where the collagen hydrogel layer closely interacts with the PCL/PLCL fibers (Population 2), resulting in locally increased stiffness. The highest modulus values are most likely associated with rigid fibers within the construct, such as collagen fibre bundles or possibly residual impurities resulting from the collagen isolation from porcine skin.

## Discussion

Full-thickness skin equivalents composed of either scaffold-free or scaffold-based organotypic cell cultures are gathering increasing interest for their potential to cover large skin defects and, most importantly, for representing a way to conduct preclinical research in an animal-free setting. Despite the availability of various commercially produced 3D models (provided by Episkin, MatTek, Phenion, etc.), the lack of a functional capillary network and skin appendages remains a major unresolved challenge in skin tissue engineering. Avascular tissue-engineered constructs with thicknesses of 100 to 200 µm are still within the oxygen diffusion limit.^23^ However, upon implantation, such constructs suffer from insufficient oxygen supply and a lack of nutrients and growth factors due to their non-ideal proximity to the vasculature, which can lead to cell apoptosis and necrosis during wound healing.^24,25^ The diffusion distance from a capillary wall to the nearest cell in physiological conditions is typically not more than 10 µm.^26^ Introducing pre-vascularization into the skin construct enables inosculation of the construct and preexisting capillaries in the host tissue. As demonstrated by Miyazaki et al.*,*^27^ a pre-vascularized, 320 µm-thick scaffold-free skin model composed using the ECM-cell coating technique, incorporating HUVECs, human fibroblasts, and keratinocytes, promoted neovascularization and was fully perfused at 7 days after implantation in mice.

Scaffold-based models offer an advantage for scaling up to replicate the native millimeter-scale thickness of the skin and the proportions of the dermal to epidermal layers, while maintaining better structural integrity during handling. In the present study, we induced the formation of a capillary-like network by leveraging the synergistic effect of ADSCs and HUVECs in order to pre-vascularize a scaffold system combining an ECM-like environment of collagen hydrogel reinforced by a fibrin-coated nanofibrous PCL/PLCL membrane. The biocompatibility of PCL and its copolymers for biomedical applications is well established, as has been demonstrated in various studies.^14,28–30^ The nanomembrane itself serves as an important structural element of our construct to minimize the contraction of the hydrogel caused by traction forces generated by HUVEC cells embedded within, as we showed in Bacakova and Pajorova et al.^22^ It helps balance the mechanical stability and the relatively low stiffness of the collagen hydrogel, both of which are necessary for proper cell spreading, growth, and migration. Additionally, the porous membrane improved the polymerization of the collagen hydrogel by facilitating fluid drainage during neutralization. Last but not least, the nanofibrous membrane provides a substrate for the adhesion and proliferation of ADSCs.

Mural cells, i.e. smooth muscle cells^31^ or pericytes,^32^ were observed to surround and stabilize endothelial cells, which positively stimulate angiogenesis. Pericytes produce proangiogenic growth factors that guide endothelial cells into capillary formation.^33^ The primary ADSC culture used in our study was selected due to its high positivity (76.1%) for the adhesion molecule CD146 (laminin alpha 4 receptor), which is considered a specific marker of blood endothelial cells and human dermal pericytes.^19^ CD146-positive ADSCs and mesenchymal stem cells (MSCs) in general represent a highly functional subpopulation, characterized by enhanced angiogenic, regenerative, immunomodulatory, and differentiation capabilities. These cells are often considered to have greater therapeutic potential than CD146-negative cells. CD146-positive cells are found primarily in perivascular regions and produce high levels of angiogenic and growth factors (e.g., VEGF, angiopoietin-1, prostaglandin E2). CD146 is also a marker of higher quality and stability and is used to identify “younger” or more “stem-like” cells. With increasing donor age or after repeated passaging, their number often decreases, which correlates with reduced differentiation potential.^18,20^

We further supported the formation of capillary-like structures with media supplements: VEGF-A, the most potent proangiogenic factor, and FGF2, which stimulates endothelial cell growth and migration.^34^ It has been reported that FGF2 binds fibrinogen or fibrin with high affinity and, as such, is more resistant to proteolytic degradation.^35^ Therefore, we functionalized the PCL/PLCL membrane with fibrin mesh nanocoating^15^ to act as a potential reservoir for the growth factor. FGF2 in the cell culture media bound to the fibrin-coated membrane during ADSC seeding and subsequent cultivation potentiated endothelial cell proliferation and accelerated the growth and migration of both cell types when HUVECs were added later.

A similar strategy was described by Chan et al.*,*^36^ who used ADSCs isolated from the burned skin of military patients to create a three-layered skin construct consisting of a collagen and polyethylene glycolated (PEG)–fibrin hydrogel. Their results highlighted the influence of the matrix microenvironment surrounding the cells: ADSCs in a collagen layer exhibited spindle-like morphology, maintaining their stromal phenotype, while ADSCs in a PEG-fibrin layer assumed a vascular phenotype, formed tubular capillary-like structures and eventually created a dense network.^36^ Unlike Chan et al.*,*^36^ we additionally embedded the HUVECs within the collagen hydrogel layer to guide ADSC migration and accelerate the formation of capillary-like structures. Endothelial cells and endothelial progenitor cells have been previously used in many studies on the construction of pre-vascularized skin^37,38^, which usually require the presence of other mesenchymal stem/stromal cells (i.e. MSCs isolated from bone marrow or adipose tissue) to achieve a mature vascular phenotype. For example, Sánchez-Muñoz et al.^39^ induced a spontaneous formation of capillary-like structures inside a 3D fibrin matrix scaffold when co-cultures of both ADSCs and HUVECs were present, but not when cultured separately. A similar cell setup was also used by Duttenhoefer et al.*,*^40^ who pre-vascularized a 3D polyurethane scaffold by seeding the endothelial progenitor cells with mesenchymal stem cells isolated from bone marrow. In the work of Baltazar et al.*,*^41^ a 3D printing method was used to create a pre-vascularized dermis by embedding human endothelial cells, human foreskin dermal fibroblasts, and human placental pericytes, rather than ADSCs, into a collagen type I scaffold. The human foreskin keratinocytes were then printed onto the construct to form an epidermis. In the study by Zimoch et al.*,*^8^ they used human dermal microvascular endothelial cells and fibroblasts for pre-vascularization, with ADSCs present in an additional layer of their construct. Therefore, in the present skin model, we combined HUVECs embedded in a collagen hydrogel with ADSCs, which migrated into the hydrogel from the underlying fibrin-coated membrane. Using ADSCs that were not embedded in a collagen hydrogel, we observed the gradual formation of a capillary-like network, particularly at the fibrin-collagen interface, where ADSCs could directly interact with embedded HUVECs.

The successful development of pre-vascularization in the dermal layer of skin constructs is influenced by the migratory capacity and phenotypic plasticity of ADSCs, as shown by Chan et al.^36^ In the model described in the present study, the ADSCs demonstrated excellent adherence and growth on the fibrin-coated PCL/PLCL membranes. Subsequent observation revealed that their migration from the membrane into the collagen hydrogel is strongly dependent not only on its dimensionality but most importantly on the environmental influence of other cell types within the scaffold. This was documented by changes in cell morphology during co-culture and differences in the definition of the capillary-like structures formed in the construct when all three cell types were introduced. Our experimental model successfully recapitulated the initial phases of vasculogenesis, as evidenced by the formation of capillary-like structures driven by the interaction of ADSCs and HUVECs. However, the presence of differentiating hKs at a later stage of the model cultivation led to the loss of definition and a subtle regression of these structures. Such observation suggests a change in paracrine crosstalk during the differentiation and stratification of hKs, which may conflict with the process of stabilizing the pre-vasculature in the construct during our chosen cultivation period.

*In vivo* epidermis is avascular due to the secretion of anti-angiogenic factors, thrombospondins (TSP-1, TSP-2), expressed by keratinocytes in the undifferentiated basal epidermal layer.^42^ TSP-1 is known to inhibit the migration and proliferation of vascular endothelial cells, contributing to the spatial separation of the vascularized dermis underneath. For example, in murine model, transgenic TSP-1 suppressed angiogenesis and vascular remodeling during full-thickness wound-healing, even in the presence of high VEGF concentration.^43^ Similarly, as various other cell types involved vascular homeostasis and wound healing, such as endothelial cells, pericytes and vascular smooth muscle cells, keratinocytes were also reported to express VEGF receptors (specifically VEGFR-1),^44^ making them both sources and targets for VEGF. The binding of VEGF to keratinocyte receptors can effectively reduce its local concentration in the vicinity of HUVECs and ADSCs in the upper part of our hydrogel model when it is cultivated submerged. Additionally, cultivation of the model at an air-liquid interface leads to changes in the diffusion gradients of oxygen and nutrients. During differentiation, keratinocytes alter their metabolism towards oxidative phosphorylation.^45^ A vertical gradient of nutrients from the culture media diffusing through the hydrogel, leads to competition between the keratinocytes exposed to normoxic conditions and the HUVECs and ADSCs within the hydrogel, potentially disrupting the stabilization of capillary-like structures. We hypothesize that this effect could be mitigated by prolonging the ADSC-HUVEC co-culture. Allowing more time for the ADSCs to migrate from the PCL/PLCL membrane and interact with HUVECs before adding hKs may yield more stable structures. This assumption is consistent with our observation that tubular pre-capillary structures emerged on the surface of the hydrogel in the absence of keratinocytes, forming openings through which endothelial cells spread across the entire surface to form a confluent layer, resembling the *tunica intima* of a blood vessel wall. This led us to the idea that our original skin construct could also be converted into a vascular wall model if we could differentiate adipose-derived stem cells (ADSCs) into smooth muscle cells. We achieved this level of differentiation in previous studies using fibrin gel^46^ or modified polylactide substrates.^47^ Furthermore, CD146-positive cells in adipose tissue and other tissues (e.g., bone marrow) are often identified as pericytes, which are inherently primed for vascular lineage differentiation and have increased contractile and myogenic potential.^48,49^

Physiological exposure of the apical top layer of the skin construct to air via an air-liquid interface (ALI) is essential for the terminal differentiation of keratinocytes.^50,51^ In our study, the keratinocyte layers cultivated at ALI consistently exhibited a larger area covered by differentiated keratinocytes positive for cytokeratin 10, and a higher thickness of the keratinocyte layer as compared to keratinocytes cultivated in immersed conditions. Observed epidermal layer, which reached 58.33 ± 2.26 µm after seven days of cultivation at ALI is within the dimensions of epidermis of human skin (50–100 µm), underlying the stratum corneum.^52^

The mechanical properties of our skin model scaffold reflect the close interaction between the underlying PCL/PLCL membrane used for reinforcement and the collagen hydrogel. The lowest modulus values, in the tens of kPa, are typically reported for collagen hydrogels depending on concentration and crosslinking.^53^ The intermediate population at hundreds of kPa is consistent with mechanically reinforced substitutes in which collagen matrices are combined with synthetic polymers.^54^ The intermediate modulus values range overlap with reported mechanical properties of human skin, which can range from several hundred kPa to a few MPa depending on anatomical site and testing configuration.^55^ The highest modulus values of MPa are consistent with reported mechanical properties of electrospun PCL-based fibers and mats, which commonly exhibit elastic moduli in the low-MPa range.^56^ The MPa values align with the upper end of the range of whole-skin mechanical properties reported in *in vivo* and *ex vivo* studies,^55,57^ suggesting that the rigid fibrous phase provides structural reinforcement comparable to that of native skin under load.

## Conclusions and further perspectives

The present tri-culture hydrogel model replicates the hallmarks of a native skin tissue equivalent, including both a pre-vascularized dermis and a stratified epidermal layer. The structural integrity of this model relies on a 3D collagen type I hydrogel reinforced with a fibrin-coated nanofibrous PCL/PLCL membrane, which effectively prevents the mechanical deformation typically caused by cellular traction forces. Mechanical characterization confirmed the composite nature of the scaffold, yielding three distinct modulus ranges (∼17 kPa, ∼0.9 MPa, and ∼3.6 MPa) that accurately mirror the intrinsic mechanical heterogeneity of native skin. The interaction of HUVECs and ADSCs within the hydrogel positively drives dermal pre-vascularization, with ADSCs demonstrating the capacity to substitute the stabilizing function of pericytes, enabling the formation of capillary-like structures. Integration of the keratinocyte layer into the tri-culture system, upon raising the model to the air-liquid interface, enhanced keratinocyte differentiation and promoted epidermal stratification; however, it also led to a subtle regression in the definition of the underlying capillary-like structures. Overall, this reinforced hydrogel model provides a mechanically stable, biologically active, and pre-vascularized skin equivalent that closely mimics the layers of dermal capillaries and epidermis. The platform offers significant potential for applications in regenerative medicine, wound-healing research, and the development of advanced, multi-layered *in vitro* skin models. Moreover, in the absence of keratinocytes, our construct has the potential to serve as a model of the blood vessel wall.

## Data Availability

The datasets generated and analyzed during the current study are available in the Zenodo repository at https://doi.org/10.5281/zenodo.21218676.
